# Diagnostic performance of Ziehl-Neelsen staining and Auramine-Rhodamine staining techniques in the detection of pulmonary and extrapulmonary tuberculosis

**DOI:** 10.17843/rpmesp.2025.421.14062

**Published:** 2025-03-18

**Authors:** Gustavo Tapia-Sequeiros, Miguel Hueda-Zavaleta, Juan Carlos Gómez de la Torre, Arantsa Hernandez-Vargas, Claudia Barletta-Carrillo, Cinthya Flores, Cristian Piscoche, Cecilia Miranda, Ada Mendoza

**Affiliations:** 1 Faculty of Health Sciences, Private University of Tacna, Tacna, Peru. Private University of Tacna Faculty of Health Sciences Private University of Tacna Tacna Peru; 2 Diagnosis, treatment, and research of infectious and tropical diseases, Private University of Tacna, Tacna, Peru. Private University of Tacna Diagnosis, treatment, and research of infectious and tropical diseases Private University of Tacna Tacna Peru; 3 Daniel Alcides Carrión Hospital III - EsSalud, Calana, Tacna, Peru. Daniel Alcides Carrión Hospital III - EsSalud Calana Tacna Peru; 4 Sequence Reference Lab, San Isidro, Lima, Peru. Sequence Reference Lab San Isidro Lima Peru

**Keywords:** Tuberculosis, Mycobacterium tuberculosis, Microbiological Techniques, Rapid Diagnostic Tests, Peru

## Abstract

**Objective.:**

To determine the diagnostic performance of Ziehl-Neelsen and Auramine-Rhodamine staining for pulmonary and extrapulmonary tuberculosis.

**Materials and methods.:**

This was a cross-sectional diagnostic test study. We used a database of processed samples from patients with suspected pulmonary and extrapulmonary tuberculosis in a private laboratory in Peru between 2011 and 2022. Ziehl-Neelsen staining and Auramine-Rhodamine staining were the index tests. The reference tests were Xpert MTB/RIF and Xpert Ultra. The receiver operating characteristics and area under the curve (AUC/ROC) were calculated to determine the diagnostic performance of each staining technique.

**Results.:**

We analyzed 908 samples processed by Ziehl-Neelsen staining and 623 samples by Auramine-Rhodamine staining, most were lung tissue samples. Using the Xpert MTB/RIF as a reference test, the Ziehl-Neelsen staining obtained an AUC=0.72, a sensitivity of 44.2% and specificity of 99.3%; and Auramine-Rhodamine staining showed an AUC=0.73, a sensitivity of 46.8% and specificity of 100%. Considering the Xpert Ultra test as a reference, the diagnostic performance for Ziehl-Neelsen showed an AUC=0.63 with a sensitivity of 26.9% and specificity of 98.5%; and an AUC=0.64 with a sensitivity of 30% and specificity of 98.2% for the Auramine-Rhodamine staining.

**Conclusion.:**

The diagnostic performance for both staining techniques is fair, and decreases when taking Xpert Ultra as a reference. New diagnostic alternatives with adequate performance are needed to detect tuberculosis.

## INTRODUCTION

Tuberculosis is one of the most significant public health problems worldwide. It is estimated that a quarter of the world’s population is infected with *Mycobacterium tuberculosis*, but only 5-10% develop clinical disease. Tuberculosis ranks second in infectious disease mortality worldwide ^1^. In Latin America, there are between 200,000 and 300,000 cases per year, and Peru has the second highest incidence of cases ^2^.

Early diagnosis using rapid, accurate, and accessible methods is essential for tuberculosis control. Mycobacterial culture was the gold standard method for tuberculosis, but the long time it takes to obtain results hinders timely diagnosis ^3^. Currently, the World Health Organization (WHO) recommends the use of molecular tests such as Xpert MTB/RIF, Xpert Ultra, and Truenat MTB as the initial method ^4^. These molecular tests have high diagnostic performance ^5^^,^^6^. However, in some low- and middle-income countries, conventional methods such as smear microscopy continue to be used due to their lower maintenance costs ^7^.

Smear microscopy can be performed using staining techniques such as Ziehl-Neelsen or Auramine-Rhodamine; however, these techniques lack good diagnostic performance for both pulmonary and extrapulmonary tuberculosis ^8^. Ziehl-Neelsen staining has a sensitivity and specificity ranging from 60 to 80%, using Lowenstein-Jensen culture as a reference ^9^. However, this parameter may decrease if molecular methods such as Xpert MTB/RIF are used as a reference, where sensitivity is reduced to 54.8%, highlighting the shortcomings of this smear technique ^8^.

To accurately determine the diagnostic performance of smear microscopy techniques, it is necessary to compare them with reference methods recommended by the WHO ^1^. Despite the high prevalence of tuberculosis in Peru, the evidence available to evaluate the performance of early diagnostic techniques is limited. The continued use of inaccurate techniques leads to unidentified cases of tuberculosis, increasing the spread of the disease and patient morbidity and mortality. For this reason, this study aimed to determine the diagnostic performance of Ziehl-Neelsen and Auramine-Rhodamine staining for identifying pulmonary and extrapulmonary tuberculosis, using molecular tests as a reference.

**KEY MESSAGES**

Motivation for the study. Peru has a high prevalence of tuberculosis, therefore, it is necessary to use diagnostic methods with high sensitivity and specificity to adequately identify cases and provide timely treatment.

Main findings. Our results indicate that, when comparing the smear microscopy techniques commonly used in Peru with the molecular tests recommended by the WHO, direct microscopy techniques show low sensitivity, less than 50%, but high specificity, greater than 95%.

Implications for public health. It is necessary to evaluate the implementation and associated costs of new rapid diagnostic alternatives that have adequate capacity to identify the majority of tuberculosis cases.

## MATERIALS AND METHODS

### Study design and setting

A cross-sectional diagnostic study was conducted using the database of samples processed by the ROE Clinical Laboratory from January 1, 2011, to December 31, 2022.

The ROE Clinical Laboratory is a private laboratory with offices in three cities in Peru: Lima, Arequipa, and Cajamarca. For this study, only samples processed at the offices located in Metropolitan Lima were considered. The laboratory has ISO 9001 and NTP ISO 15189 quality certifications. Its facilities perform more than 2,500 specialized clinical analyses, including smear microscopy and molecular tests for the diagnosis of tuberculosis. Between 2011 and 2018, the clinical laboratory used Xpert MTB/RIF for the processing of samples with suspected tuberculosis. Starting in 2019, this was replaced by Xpert Ultra as the only molecular test.

We followed the recommendation of the “Standards for Reporting Diagnostic Accuracy Studies” (STARD) guideline for reporting the results of this study ^10^.

### Participants

The study included records of samples from patients ≥ 18 years of age with clinical or radiological suspicion of pulmonary or extrapulmonary tuberculosis who had been previously evaluated by a physician at their respective healthcare facility and who came to the private clinical laboratory with a signed medical prescription. These samples underwent at least one smear microscopy technique (Ziehl-Neelsen staining +/- Auramine-Rhodamine staining), and a molecular test (Xpert MTB/RIF or Xpert Ultra) to confirm or rule out tuberculosis. Pulmonary samples were obtained by bronchoalveolar lavage (BAL), bronchial aspiration, or sputum. Extrapulmonary samples were cerebrospinal fluid (CSF), urine, pleural fluid, ascites fluid, lymph node biopsy, soft tissue and parenchymal biopsy (breast, pleura, endometrium, liver, muscle tissue), soft tissue and parenchymal abscess (breast, kidney, muscle tissue), and others (pericardial fluid, bone tissue, synovial fluid, gastric aspirate). Records with incomplete data or indeterminate results were excluded.

The statistical power was calculated for the sample size of the processed samples, taking as reference a study that reported a sensitivity of 54.8% for Ziehl-Neelsen staining and 84.5% for Auramine-Rhodamine staining ^8^. With these values, we obtained a statistical power greater than 80%.

### Index test

In this study, there were two index tests: Ziehl-Neelsen staining and Auramine-Rhodamine staining. Sample processing was performed by trained personnel from a private clinical laboratory, following the “Manual of Bacilloscopy Procedures for the Bacteriological Diagnosis of Tuberculosis” of the Peruvian Ministry of Health (MINSA) ^11^. Pulmonary and extrapulmonary samples were processed within 24 hours of collection.

In the Ziehl-Neelsen stain, the smears were stained with 0.3% phenol fuchsin and left to stain for 5 minutes. Next, decolorization was performed with acid alcohol solution for 3 minutes, followed by rinsing with water to remove excess stain. After that, background staining was performed with 0.1% methylene blue and left to dry at room temperature. The smears were examined by direct microscopy at 100X magnification to identify acid-fast bacilli (AFB).

In Auramine-Rodamine staining, the smear was stained with fluorescent dye and left to act for 15 to 20 minutes. Decolorization was performed with acid-alcohol for 1 to 3 minutes, followed by rinsing with water and allowing excess dye to drain. The background staining was performed with 0.5% potassium permanganate for 1 minute and left to dry at room temperature, protecting it from ultraviolet light. The smears were examined by fluorescence microscopy with an LED lamp at magnifications of 200X and 400X.

The results of the processed samples were used to construct a database by trained laboratory personnel. We followed the recommendations of the MINSA Technical Standard for Tuberculosis to establish the criteria for identifying a positive result in smear microscopy techniques, ^12^. A positive Ziehl-Neelsen stain result was considered to be the presence of 1 to 9 AFB bacilli in 100 observed fields (paucibacillary sample), 10 to 99 AFB in 100 observed fields, 1 to 10 AFB per field in 50 observed fields, or 10 AFB per field in 20 observed fields. On the other hand, the Auramine-Rhodamine stain was considered positive if, with a 200X magnification, there were 5 to 49 AFB in a line (paucibacillary sample) or more than 3 AFB per field. With a 400X magnification, a positive result was considered if there were between 3 and 24 AFB in a line (paucibacillary sample) or more than 1 AFB per field.

### Reference tests

The reference tests were Xpert MTB/RIF and Xpert Ultra. Sample processing was performed according to the manufacturer’s guidelines (Cepheid Inc., Sunnyvale, CA, USA) ^13^^,^^14^. The samples were stored at a temperature of 2 to 8 °C. The samples were transferred to 15 ml tubes together with the reagent provided by the manufacturer. The tubes were then closed and shaken vigorously 10 to 20 times to ensure adequate sample integration. Subsequently, 500 µL to 2 mL were aspirated and placed in the Xpert cartridge, which was inserted into the GeneXpert device for automated processing.

The difference between Xpert MTB/RIF and Xpert MTB Ultra is the time and ability to detect various *Mycobacterium tuberculosis* genes. A positive result for Xpert MTB/RIF was indicated by the detection of the ropβ gene and its mutations at the cycle threshold (Ct), while a positive result for Xpert Ultra was indicated by the detection of the IS6110 and IS1081 gene sequences, independent of rifampicin resistance ^15^.

### Statistical analysis

The database was exported to Microsoft Excel and then analyzed using the statistical program Stata version 17. Categorical variables were presented using absolute and relative frequencies. The age of the patients was presented using the median and interquartile range (IQR) due to the skewed distribution of the data. Differences in sample characteristics were assessed using the Chi-square test or Fisher’s exact test for categorical variables and the Mann-Whitney U test for the age variable. For the diagnostic performance analysis, we constructed double-entry tables to calculate the prevalence, sensitivity, specificity, positive predictive value (PPV), negative predictive value (NPV), positive likelihood ratio (LR+) and negative likelihood ratio (LR-) with their respective 95% confidence intervals (95% CI). Prevalence was calculated as the proportion of tuberculosis cases defined as samples confirmed by a molecular test (Xpert MTB/RIF or Xpert Ultra) in relation to the total number of evaluated samples. Finally, the receiver operating characteristic (ROC) curve was plotted to calculate the area under the curve (AUC) for each smear technique ^16^.

### Ethical considerations

The protocol for this study was approved by the Ethics Committee of the Faculty of Health Sciences of the Private University of Tacna (UPT) under code FACSA-CEI/081-04-2024. We requested informed consent exemption due to the observational and retrospective nature of the study. This protocol was registered on the Health Research Projects (PRISA) platform with code EI00000003212, as it addresses tuberculosis in Peru. In addition, we obtained permission from the management of the ROE clinical laboratory to access the tuberculosis database. The anonymity of each individual was guaranteed during data collection and cleaning.

## RESULTS

**Figure 1:**
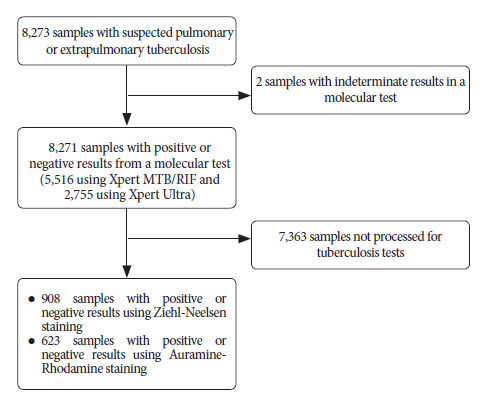
Flow chart of the final number of samples with suspected tuberculosis analyzed in this study.

A total of 908 samples were collected from patients with clinical suspicion of tuberculosis. All samples were processed using Ziehl-Neelsen staining, and 623 of them were also processed using Auramine-Rhodamine staining (Figure 1). Of the total samples processed, 530 were tested using Xpert MTB/RIF and 378 using Xpert Ultra.

The samples processed by Xpert MTB/RIF came from patients with a median age of 60 (IQR 42-74) years. Of these samples, 78.3% were pulmonary, mostly collected by bronchoalveolar lavage (38.1%). The remaining 21.7% were extrapulmonary samples, mainly urine (27.8%). We found positive results for tuberculosis in 95 (17.9%) samples, of which 80 were pulmonary samples and 15 were extrapulmonary. Likewise, of the total Xpert MTB/RIF-positive samples, 53 (55.8%) were not detected by Ziehl-Neelsen staining and 41 (53.2%) were not detected by Auramine-Rhodamine staining (Table 1).

**Table 1:** General characteristics of samples processed with Xpert MTB/RIF (n=530).

Characteristics		n (%)	Xpert MTB/RIF		
			Positive n=95 (17.9%)	Negative n=435 (82.1%)	p-value ^a^
Age (n=395) median (IQR)		60 (42-74)	46.5 (35-66)	61 (46-75)	<0.001
Gender (n=466)					0.277
	Female	247 (53.0)	39 (15.8)	208 (84.2)	
	Male	219 (47.0)	43 (19.6)	176 (80.4)	
Origin of the sample					0.123
	Pulmonary	415 (78.3)	80 (19.3)	335 (80.7)	
	Extrapulmonary	115 (21.7)	15 (13.0)	100 (87.0)	
Lung samples					0.337
	Bronchoalveolar lavage	158 (38.1)	30 (19.0)	128 (81.0)	
	Bronchial aspiration	105 (25.3)	25 (23.8)	80 (76.2)	
	Sputum	152 (36.6)	25 (16.5)	127 (83.5)	
Extrapulmonary samples					0.026
	CSF	21 (18.3)	3 (14.3)	18 (85.7)	
	Urine	31 (27.8)	1 (3.1)	31 (96.9)	
	Pleural fluid	17 (14.8)	2 (11.8)	15 (88.2)	
	Ascitic fluid	4 (3.5)	0 (0.0)	4 (100.0)	
	Lymph node biopsy	5 (4.3)	3 (60.0)	2 (40.0)	
	Soft tissue and parenchyma biopsy	11 (9.6)	1 (9.1)	10 (90.9)	
	Soft tissue and parenchyma abscess	12 (10.4)	1 (8.3)	11 (91.7)	
	Other	13 (11.3)	4 (30.8)	9 (69.2)	
Ziehl-Neelsen staining (n=530)					< 0.001
	Positive	45 (8.5)	42 (93.3)	3 (6.7)	
	Negative	485 (91.5)	53 (10.9)	432 (89.1)	
Auramine-Rhodamine staining (n=395)					< 0.001
	Positive	36 (9.1)	36 (100.0)	0 (0.0)	
	Negative	359 (90.9)	41 (11.4)	318 (88.6)	

IQR: interquartile range, CSF: cerebrospinal fluid

a The p-value for categorical variables was calculated using the chi-square test or Fisher’s exact test (in extrapulmonary samples). The p-value for the patient age variable was calculated using the Mann-Whitney U test.

The samples processed with Xpert Ultra came from patients with a median age of 56 (IQR 37-74) years, more than half of whom were male (54.3%). Of these processed samples, 78.3% were of pulmonary origin, mainly from bronchial aspiration (40.9%). There were 104 positive results (27.5%), of which 91 were of pulmonary origin and 13 were of extrapulmonary origin. Similarly, of the total Xpert Ultra-positive samples, 76 (73.1%) were not identified by Ziehl-Neelsen staining and 42 (70.0%) were not detected by Auramine-Rhodamine staining (Table 2).

**Table 2:** General characteristics of samples processed with Xpert Ultra (n=378).

Characteristics		n (%)	Xpert Ultra		
			Positive n=104 (27.5%)	Negative n=274 (72.5%)	p-value ^a^
Age (n=335), median (IQR)		56 (37-74)	48 (30-69)	59 (41-75)	0.006
Gender (n=328)					0.448
	Female	150 (45.7)	39 (26.0)	111 (74.0)	
	Male	178 (54.3)	53 (29.8)	125 (70.2)	
Origin of the sample					0.008
	Pulmonary	296 (78.3)	91 (30.7)	205 (69.3)	
	Extrapulmonary	82 (21.7)	13 (15.8)	69 (84.2)	
Lung samples					0.213
	Bronchoalveolar lavage	85 (28.7)	32 (37.7)	53 (62.3)	
	Bronchial aspiration	121 (40.9)	36 (29.8)	85 (70.2)	
	Sputum	90 (30.4)	23 (25.6)	67 (74.4)	
Extrapulmonary samples					0.192
	CSF	6 (7.3)	0 (0.0)	6 (100.0)	
	Urine	20 (24.4)	3 (15.0)	17 (85.0)	
	Pleural fluid	20 (24.4)	1 (5.0)	19 (95.5)	
	Ascitic fluid	4 (4.9)	0 (0.0)	4 (100.0)	
	Lymph node biopsy	7 (8.5)	3 (42.9)	4 (57.1)	
	Soft tissue and parenchyma biopsy	5 (6.1)	1 (20.0)	4 (80.0)	
	Soft tissue and parenchyma abscess	11 (13.4)	2 (18.2)	9 (81.8)	
	Other	9 (11.0)	3 (33.3)	6 (66.7)	
Ziehl-Neelsen staining (n=378)					< 0.001
	Positive	32 (8.5)	28 (87.5)	4 (12.5)	
	Negative	346 (91.5)	76 (22.0)	270 (78.0)	
Auramine-Rhodamine staining (n=228)					< 0.001
	Positive	21 (9.2)	18 (85.7)	3 (14.3)	
	Negative	207 (90.8)	42 (20.3)	165 (79.7)	

IQR: interquartile range, CSF: cerebrospinal fluid

a The p-value for categorical variables was calculated using the chi-square test or Fisher’s exact test (in extrapulmonary samples). The p-value for the patient age variable was calculated using the Mann-Whitney U test.

When comparing Ziehl-Neelsen staining with Auramine-Rhodamine staining, we found good concordance between the two smear techniques (Cohen’s Kappa index = 0.990). We observed that only one sample that was positive with Ziehl-Neelsen staining was not detected with Auramine-Rhodamine staining. On the other hand, moderate concordance was found when comparing Ziehl-Neelsen staining (Cohen’s Kappa index = 0.548) and Auramine-Rhodamine staining (Cohen’s Kappa index = 0.586) with Xpert MTB/RIF, and weak agreement when comparing both smear techniques with Xpert Ultra (Cohen’s kappa index = 0.324 and 0.357) (Table 3).

**Table 3:** Concordance analysis of smear testing techniques compared with Xpert MTB/RIF and Xpert Ultra.

Test comparison	Overall concordance	Cohen’s Kappa Index	Standard error	p-value
Ziehl-Neelsen staining vs. Auramine-Rhodamine staining	99.8	0.990	0.040	< 0.001
Ziehl-Neelsen staining vs. Xpert MTB/RIF	89.4	0.548	0.039	< 0.001
Ziehl-Neelsen staining vs. Xpert Ultra	78.8	0.324	0.041	< 0.001
Auramine-Rhodamine staining vs. Xpert MTB/RIF	89.6	0.586	0.046	< 0.001
Auramine-Rhodamine staining vs. Xpert Ultra	80.3	0.357	0.055	< 0.001

Using Xpert MTB/RIF as a reference, we found that, for the total sample, the diagnostic accuracy of Ziehl-Neelsen staining (AUC=0.72; 95% CI: 0.67-0.77) and Auramine-Rhodamine staining (AUC=0.73; 95% CI: 0.68-0.79) were similar (Supplementary Material 1A and 1B).

We also reported a sensitivity of 44.2% (95% CI: 34.0-54.8) and 46.8% (95% CI: 35.3-58.5), and specificity of 99.3% (95% CI: 98.0-99.9) and 100% (95% CI: 98.8-100), respectively (Supplementary Material 2A). A difference in diagnostic performance was found in extrapulmonary samples, with Auramine-Rhodamine staining showing better diagnostic performance (AUC=0.71; 95% CI: 0.52-0.91) compared to Ziehl-Neelsen staining (AUC=0.65; 95% CI: 0.53-0.78) (Table 4).

**Table 4:** Diagnostic performance of smear microscopy techniques for pulmonary and extrapulmonary tuberculosis compared with Xpert MTB/RIF.

	Prevalence % (95%CI)	Sensitivity % (95%CI)	Specificity % (95%CI)	PPV (95%CI)	NPV (95%CI)	LR + (95%CI)	LR - (95%CI)	AUC/ROC (95%CI)
Total samples								
Ziehl-Neelsen stain	17.9 (14.8-21.5)	44.2 (34.0-54.8)	99.3 (98.0-99.9)	93.3 (81.7-98.6)	89.1 (86.0-91.7)	64.1 (20.3-202.5)	0.6 (0.5-0.7)	0.72 (0.67-0.77)
Auramine-Rhodamine stain	19.5 (15.7-23.7)	46.8 (35.3-58.5)	100 (98.8-100)	100 (90.3-100)	88.6 (84.8-91.7)	-	0.5 (0.4-0.7)	0.73 (0.68-0.79)
Lung samples								
Ziehl-Neelsen stain	19.3 (15.6-23.4)	46.3 (35.0-57.8)	100 (98.9-100)	100 (90.5-100)	88.6 (85.0-91.6)	-	0.5 (0.4-0.7)	0.73 (0.68-0.79)
Auramine-Rhodamine stain	18.8 (15.0-23.2)	47.1 (35.1-59.4)	100 (98.8-100)	100 (89.4-100)	89.1 (85.3-92.2)	-	0.5 (0.4-0.7)	0.74 (0.68-0.79)
Extrapulmonary samples								
Ziehl-Neelsen stain	13.0 (7.5-20.6)	33.3 (11.8-61.6)	97.0 (91.5-99.4)	62.5 (24.5-91.5)	90.7 (83.5-95.4)	11.1 (2.9-41.8)	0.7 (0.5-0.8)	0.65 (0.53-0.78)
Auramine-Rhodamine stain	30.4 (13.2-52.9)	42.9 (9.9-81.6)	100 (79.4-100)	100 (29.2-100)	80.0 (56.3-94.3)	-	0.6 (0.3-1.1)	0.71 (0.52-0.91)

AUC: area under the curve; ROC: receiver operating characteristics; PPV: positive predictive value; NPV: negative predictive value; LR: likelihood ratio; 95% CI: 95% confidence interval.

Using Xpert Ultra as a reference, the diagnostic accuracy for total samples was AUC=0.63 (95% CI: 0.58-0.67) for Ziehl-Neelsen staining (Supplementary Material 1C), with a sensitivity and specificity of 26.9% (95% CI: 18.7-36.5) and 98.5% (95% CI: 96.3-99.6), respectively (Supplementary Material 2B). The diagnostic accuracy of Auramine-Rodamine staining was AUC=0.64 (95% CI: 0.58-0.70) with a sensitivity of 30% (95% CI: 18.8-43.2) and specificity of 98.2% (95% CI: 94.9-99.6) (Supplementary Material 1 D and 2B). We found a slight difference in diagnostic performance in lung samples between Auramine-Rodamine staining (AUC=0.65; 95% CI: 0.59-0.71) and Ziehl-Neelsen staining (AUC=0.63; 95% CI: 0.58-0.68) (Table 5).

**Table 5:** Diagnostic performance of smear microscopy techniques for pulmonary and extrapulmonary tuberculosis compared with Xpert Ultra.

		Prevalence % (95%CI)	Sensitivity % (95%CI)	Specificity % (95%CI)	PPV (95%CI)	NPV (95%CI)	LR + (95%CI)	LR - (95%CI)	AUC/ROC (95%CI)
Total samples									
	Ziehl-Neelsen stain	27.5 (23.2-32.3)	26.9 (18.7-36.5)	98.5 (96.3-99.6)	87.5 (71.0-96.5)	78.0 (73.3-82.3)	18.4 (6.6-51.3)	0.7 (0.7-0.8)	0.63 (0.58-0.67)
	Auramine-Rhodamine stain	26.3 (20.7-32.5)	30.0 (18.8-43.2)	98.2 (94.9-99.6)	85.7 (63.7-97.0)	79.7 (73.6-85.0)	16.8 (5.1-55.0)	0.7 (0.6-0.8)	0.64 (0.58 -0.70)
Lung samples									
	Ziehl-Neelsen stain	30.7 (25.5-36.3)	27.5 (18.6-37.8)	98.5 (95.8-99.7)	89.3 (71.8-97.7)	75.4 (69.8-80.4)	18.8 (5.8-60.6)	0.7 (0.7-0.8)	0.63 (0.58-0.68)
	Auramine-Rhodamine stain	26.5 (20.7-32.9)	31.6 (19.9-45.2)	98.1 (94.6-99.6)	85.7 (63.7-97.0)	79.9 (73.6-85.3)	16.6 (5.1-54.4)	0.7 (0.6-0.8)	0.65 (0.59-0.71)
Extrapulmonary samples									
	Ziehl-Neelsen stain	15.9 (8.7-25.6)	23.1 (5.0-53.8)	98.6 (92.2-100.0)	75.0 (19.4-99.4)	87.2 (77.7-93.7)	15.9 (1.8-141.5)	0.8 (0.6-1.1)	0.61 (0.49 -0.73)
	Auramine-Rhodamine stain	-	-	-	-	-	-	-	-

AUC: area under the curve; ROC: receiver operating characteristics; PPV: positive predictive value; NPV: negative predictive value; LR: likelihood ratio; 95% CI: 95% confidence interval

a Only 13 extrapulmonary samples were processed with Auramine-Rhodamine, all of which were negative for extrapulmonary TB.

## DISCUSSION

The diagnostic performance of Ziehl-Neelsen and Auramine-Rhodamine staining was evaluated using molecular tests recommended by the WHO (Xpert MTB/RIF and Xpert Ultra) as a reference. Our findings indicate that both smear microscopy techniques have moderate diagnostic capability in identifying tuberculosis cases.

Diagnostic performance may vary depending on the reference standard used. Mycobacterial culture was the gold standard in previous studies on tuberculosis diagnosis, with good discriminatory power in both smear techniques (AUC=89.7 and 90.3) and a sensitivity greater than 60% ^17^. However, the disadvantages of culture, such as the waiting time for mycobacterial growth (2 to 3 weeks) and the need for biosafety level III laboratories to analyze samples of microorganisms that cause serious diseases, hinder its use as a reference method ^18^^,^^19^. Therefore, it is necessary to establish a reference standard that overcomes these evident barriers, with the use of molecular tests being a feasible option.

We observed found consistent diagnostic performance in lung samples for both smear techniques, with a slight advantage in the discriminatory capacity of Auramine-Rhodamine staining over Ziehl-Neelsen staining. These results are consistent with those of Dzodanu *et al*., who highlighted the greater diagnostic accuracy of fluorescence techniques ^8^. These techniques allow rapid identification of bacilli in a dark field, reducing the time needed to report results ^20^.

Another important aspect of the study by Dzodanu *et al*. is the higher diagnostic performance observed in both smear techniques (AUC=0.92 and 0.77) compared to our results ^8^. This discrepancy becomes more evident when analyzing the results obtained with a different reference standard. While Dzodanu *et al*. used Xpert MTB/RIF, we used Xpert Ultra. According to previous research, Xpert Ultra is the molecular test with the best sensitivity (between 86% and 100%) and specificity (between 89% and 99%), and should therefore be considered the gold standard for pulmonary tuberculosis diagnosis studies ^5^.

We found that Ziehl-Neelsen and Auramine-Rhodamine stains had moderate discriminatory power in extrapulmonary samples but low sensitivity of 33.3% and 42.9%, respectively. The sensitivity and specificity of smear microscopy techniques are generally lower than those of molecular tests ^9^. Xpert MTB/RIF and Xpert Ultra may be useful for diagnosing extrapulmonary tuberculosis; however, diagnostic parameters vary depending on the type of sample ^21^. Both molecular methods were reported to have variable sensitivity for cerebrospinal fluid samples (71% and 89%), pleural fluid (50% and 75%), and lymph node aspirates (82% and 70%); while specificity was over 85% in all three sample types ^22^. Therefore, it is necessary to choose the diagnostic method based on the type of extrapulmonary sample to be evaluated.

Our results show that smear microscopy techniques have inadequate capacity to diagnose tuberculosis. In many low- and middle-income countries, smear microscopy continues to be used as the initial diagnostic method ^23^. The main disadvantage of these conventional techniques is their poor sensitivity, which prevents the correct identification of all patients with pulmonary or extrapulmonary tuberculosis, producing a high percentage of false negatives. The WHO recommends the use of molecular tests that have better diagnostic performance, such as Xpert MTB/RIF or Xpert Ultra. However, their maintenance costs limit their implementation in several countries ^24^. An alternative is the combined use of smear microscopy techniques with molecular methods, where only those samples with negative results in smear microscopy techniques can be processed by molecular tests ^25^. However, the limited number of public laboratories in Peru would delay diagnosis.

Another alternative is the use of diagnostic algorithms. A study conducted in Lima, Peru, reported that the combination of chest X-ray followed by a molecular test achieved a sensitivity of 68%, compared to the approach based on symptomatic evaluation followed by smear microscopy, which had a sensitivity of only 23% ^26^. Algorithms that exclude clinical and radiographic evaluation and rely solely on bacteriological tests with low diagnostic capacity, such as smear microscopy, may be perceived as less costly. However, inadequate diagnosis and lack of timely treatment can lead to higher costs for the health system in the long term ^27^.

Likewise, pooled sample processing is an accessible alternative for implementing molecular testing. Analysis of pooled samples using Xpert MTB/RIF or Xpert Ultra was found to have a sensitivity of 95% and a specificity of 97.1%, as well as achieving up to 96% concordance with the results obtained from processing individual samples ^28^. Similarly, pooled sample processing is less expensive than individual sample analysis, making it a feasible and reliable option for low- and middle-income countries such as Peru ^29^.

Our study presents some elements that should be considered when interpreting the results. On the one hand, although there are many studies comparing smear microscopy with molecular techniques such as Xpert MTB/RIF or Xpert Ultra, our work was carried out in a specific context, a private laboratory in which most of the samples were not sputum but bronchial aspirate and bronchoalveolar lavage samples, which provides information about this particular context. On the other hand, limitations such as the lack of detailed information on the sociodemographic characteristics, tuberculosis history, comorbidities, or previous treatment of the patients must be acknowledged. These factors may influence the performance of diagnostic tests, which could lead to measurement bias. The distribution of pulmonary and extrapulmonary samples was not uniform, and as mentioned above, the particular context in which the study was conducted prevents the findings from being generalized to other settings. Besides, the limited number of extrapulmonary samples prevented the determination of diagnostic accuracy for each sample type. Finally, the results may be subject to biases arising from errors in sample processing or related to the interpretation of results by laboratory personnel. Despite these limitations, the samples were collected and analyzed in a private clinical laboratory with quality certification, using modern equipment that guarantees reliable results. In addition, this study evaluates the diagnostic accuracy of smear microscopy techniques using molecular tests recommended by the WHO as a reference, which have greater diagnostic capacity than mycobacterial culture.

In conclusion, Ziehl-Neelsen staining and Auramine-Rhodamine staining were found to have moderate diagnostic performance for pulmonary and extrapulmonary samples. When comparing the results of both microscopy techniques, we found a slightly higher diagnostic capacity with Auramine-Rodamine staining. In addition, both stains showed excellent specificity but low sensitivity. Therefore, the implementation of new diagnostic alternatives should be evaluated, including the use of rapid molecular tests with adequate diagnostic performance for detecting tuberculosis, after assessing the associated costs.
