# Supplementary material for: Diagnostic performance of Ziehl-Neelsen staining and Auramine-Rhodamine staining techniques in the detection of pulmonary and extrapulmonary tuberculosis
Source: Rev Peru Med Exp Salud Publica. 2025 Mar 18;42(1):37–45. doi: 10.17843/rpmesp.2025.421.14062 (PMC12176024; doi:10.17843/rpmesp.2025.421.14062)
Supplement: Supplementary material. — Available in the electronic version of the RPMESP. [file rpmesp-42-01-14062-s001.docx]

Material suplementario

Figura 1. Características operativas del receptor (ROC) de la precisión diagnóstica de las técnicas de baciloscopia. A. Tinción Ziehl-Neelsen vs. Xpert MTB/RIF. B. Tinción Auramina-Rodamina vs. Xpert MTB/RIF. C. Tinción Ziehl-Neelsen vs. Xpert Ultra. D. Tinción Auramina-Rodamina vs. Xpert Ultra.

Figura 2. Gráfico de radar que compara el rendimiento diagnóstico de las técnicas de baciloscopia en muestras pulmonares y extrapulmonares. A. Rendimiento diagnóstico de las técnicas de baciloscopia utilizando Xpert MTB/RIF como referencia. B. Rendimiento diagnóstico de las técnicas de baciloscopia utilizando Xpert Ultra como referencia.
