# Supplementary material for: Diagnostic performance of Ziehl-Neelsen staining and Auramine-Rhodamine staining techniques in the detection of pulmonary and extrapulmonary tuberculosis
Source: Rev Peru Med Exp Salud Publica. 2025 Mar 18;42(1):37–45. doi: 10.17843/rpmesp.2025.421.14062 (PMC12176024; doi:10.17843/rpmesp.2025.421.14062)
Supplement: Translation

# Translation: Rendimiento diagnóstico de las técnicas de tinción Ziehl-Neelsen y Auramina-Rodamina en la detección de tuberculosis pulmonar y extrapulmonar

**Authors:** Gustavo Tapia-Sequeiros, Miguel Hueda-Zavaleta, Juan Carlos Gómez de la Torre, Arantsa Hernandez-Vargas, Claudia Barletta-Carrillo, Cinthya Flores, Cristian Piscoche, Cecilia Miranda, Ada Mendoza

**Affiliations:** 1 Facultad de Ciencias de la Salud, Universidad Privada de Tacna, Tacna, Perú.; 2 Diagnóstico, tratamiento e investigación de enfermedades infecciosas y tropicales, Universidad Privada de Tacna, Tacna, Perú.; 3 Hospital III Daniel Alcides Carrión - Essalud, Calana, Tacna, Perú.; 4 Sequence Reference Lab, San Isidro, Lima, Perú.

**Keywords:** Tuberculosis, Mycobacterium tuberculosis, Técnicas Microbiológicas, Prueba de Diagnóstico Rápido, Perú

## Abstract

**Objetivo.:**

Determinar el rendimiento diagnóstico de la tinción Ziehl-Neelsen y Auramina-Rodamina para tuberculosis pulmonar y extrapulmonar.

**Materiales y métodos.:**

Se realizó un estudio transversal de prueba diagnóstica. Se utilizó una base de datos de muestras procesadas de pacientes con sospecha de tuberculosis pulmonar y extrapulmonar en un laboratorio privado de Perú entre el 2011 y 2022. Las pruebas índices fueron la tinción Ziehl-Neelsen y tinción Auramina-Rodamina. Las pruebas de referencia fueron Xpert MTB/RIF y Xpert Ultra. Se calculó las características operativas del receptor y área bajo la curva (AUC/ROC) para determinar el rendimiento diagnóstico de cada técnica de baciloscopia.

**Resultados.:**

Analizamos 908 muestras procesadas por tinción Ziehl-Neelsen y 623 muestras por tinción Auramina-Rodamina siendo la mayoría de origen pulmonar. Al tomar como referencia al Xpert MTB/RIF, la tinción Ziehl-Neelsen obtuvo un AUC=0,72 con una sensibilidad del 44,2% y especificidad del 99,3%; y la tinción Auramina-Rodamina un AUC=0,73 con una sensibilidad del 46,8% y especificidad del 100%. Al tomar como referencia el Xpert Ultra, el rendimiento diagnóstico para la tinción Ziehl-Neelsen fue AUC=0,63 con una sensibilidad del 26,9% y especificidad del 98,5%; y para la tinción Auramina-Rodamina fue AUC=0,64 con una sensibilidad del 30% y especificidad del 98,2%.

**Conclusión.:**

El rendimiento diagnóstico para ambas técnicas de baciloscopia es regular, y disminuye al tomar como referencia al Xpert Ultra. Es necesario el uso de nuevas alternativas diagnosticas con un rendimiento adecuado para detectar tuberculosis.

## INTRODUCCIÓN

La tuberculosis es uno de los problemas de salud pública con mayor relevancia a nivel mundial. Se estima que la cuarta parte de la población mundial está infectada por *Mycobacterium tuberculosis*, pero solo entre el 5 y 10% desarrolla la enfermedad clínica. La tuberculosis ocupa el segundo lugar en mortalidad por causas infecciosas en el mundo ^1^. En Latinoamérica, se producen entre 200 000 y 300 000 casos al año, y Perú es el segundo país con mayor incidencia de casos ^2^.

Para el control de la tuberculosis, es esencial el diagnóstico precoz con métodos rápidos, precisos y accesibles. El cultivo de micobacterias fue el método de referencia usado para tuberculosis, pero el tiempo prolongado que tarda en obtener resultados dificultan el diagnóstico oportuno ^3^. Actualmente, la Organización Mundial de la Salud (OMS) recomienda el uso de pruebas moleculares como Xpert MTB/RIF, Xpert Ultra y Truenat MTB como método inicial ^4^. Estas pruebas moleculares tienen un alto rendimiento diagnóstico ^5^^,^^6^. Sin embargo, en algunos países de bajos y medianos ingresos se siguen utilizando métodos convencionales como la baciloscopia debido a sus menores costos de mantenimiento ^7^.

La baciloscopia puede realizarse mediante técnicas de tinción como Ziehl-Neelsen o Auramina-Rodamina; sin embargo, estas técnicas carecen de un buen rendimiento diagnóstico tanto para tuberculosis pulmonar y extrapulmonar ^8^. La tinción Ziehl-Neelsen posee una sensibilidad y especificidad variable entre 60 a 80%, tomando como referencia el cultivo Lowenstein-Jensen ^9^. Sin embargo, este parámetro puede disminuir si se toman como referencia métodos moleculares como el Xpert MTB/RIF, donde la sensibilidad se reduce al 54,8%, evidenciando la deficiencia de esta técnica de baciloscopia ^8^.

Para conocer de forma precisa el rendimiento diagnóstico de las técnicas de baciloscopia es necesario compararlo con métodos de referencia recomendados por la OMS ^1^. A pesar de la alta prevalencia de tuberculosis en Perú, la evidencia disponible para evaluar el rendimiento de técnicas de diagnóstico temprano es limitada. El uso continuo de técnicas poco precisas genera casos tuberculosis no identificados, aumentando la diseminación de la enfermedad y la morbimortalidad de los pacientes. Por tal motivo, el objetivo de este estudio fue determinar el rendimiento diagnóstico de la tinción Ziehl-Neelsen y Auramina-rodamina para identificar tuberculosis pulmonar y extrapulmonar, tomando como referencia pruebas moleculares.

**MENSAJE CLAVE**

Motivación para realizar el estudio. El Perú tiene una alta prevalencia de tuberculosis, por lo que es necesario utilizar métodos diagnósticos con alta sensibilidad y especificidad para identificar adecuadamente los casos y brindar un tratamiento oportuno.

Principales hallazgos. Nuestros resultados indican que, al comparar las técnicas de baciloscopia utilizadas habitualmente en Perú con las pruebas moleculares recomendadas por la OMS, las técnicas de microscopía directa muestran una sensibilidad baja, inferior al 50%, pero una especificidad alta, superior al 95%.

Implicancias para la Salud pública. Es necesario evaluar la implementación y los costos asociados de nuevas alternativas de diagnóstico rápido que tengan una capacidad adecuada para identificar la mayoría de casos de tuberculosis.

## MATERIALES Y MÉTODOS

### Diseño y entorno del estudio

Se realizó un estudio transversal de tipo prueba diagnóstica, utilizando como base de datos al registro de muestras procesadas por el Laboratorio Clínico ROE desde el 1 de enero del 2011 hasta el 31 de diciembre del 2022.

El Laboratorio Clínico ROE es de ámbito privado, con sedes en tres ciudades del Perú: Lima, Arequipa y Cajamarca. Para este estudio, solo se consideraron las muestras procesadas en las sedes ubicadas en Lima Metropolitana. El laboratorio cuenta con las certificaciones de calidad ISO 9001 y NTP ISO 15189. En sus instalaciones se realizan más de 2500 análisis clínicos especializados, incluyendo técnicas de baciloscopia y pruebas moleculares para el diagnóstico de tuberculosis. Entre 2011 y 2018, el laboratorio clínico utilizó Xpert MTB/RIF para el procesamiento de muestras con sospecha de tuberculosis. A partir de 2019, esta fue sustituida por el Xpert Ultra como única prueba molecular.

Para el reporte del presente estudio, se siguió la recomendación de la guía «Standards for Reporting Diagnostic Accuracy Studies» (STARD) ^10^.

### Participantes

Se incluyeron los registros de muestras de pacientes ≥ 18 años con sospecha clínica o radiológica de tuberculosis pulmonar o extrapulmonar que fueron evaluados previamente por un médico en su establecimiento de salud respectivo y que acudieron al laboratorio clínico privado con una orden médica firmada. Dichas muestras fueron sometidas al menos a una técnica de baciloscopia (tinción Ziehl-Neelsen +/- tinción Auramina-Rodamina), acompañado de una prueba molecular (Xpert MTB/RIF o Xpert Ultra) para confirmar o descartar tuberculosis. Las muestras pulmonares se obtuvieron mediante lavado broncoalveolar (LBA), aspirado bronquial o esputo. Las muestras extrapulmonares fueron de líquido cefalorraquídeo (LCR), orina, líquido pleural, líquido ascítico, biopsia de ganglio linfático, biopsia de tejidos blandos y parénquima (mama, pleura, endometrio, hígado, tejido muscular), absceso de tejidos blandos y parénquima (mama, riñón, tejido muscular), y otros (líquido pericárdico, tejido óseo, líquido sinovial, aspirado gástrico). Se excluyeron los registros con datos incompletos o resultados indeterminados.

Se calculó la potencia estadística para el tamaño muestral de las muestras procesadas, tomando como referencia un estudio que reportó una sensibilidad del 54,8% para la tinción Ziehl-Neelsen y 84,5% para la tinción Auramina-Rodamina ^8^. Con estos valores, se obtuvo una potencia estadística superior al 80%.

### Prueba índice

En este estudio hubo dos pruebas índices, que fueron la tinción Ziehl-Neelsen y la tinción Auramina-Rodamina. El procesamiento de las muestras fue realizado por personal capacitado del laboratorio clínico privado, siguiendo el «Manual de Procedimientos de Baciloscopia para el Diagnóstico Bacteriológico de la Tuberculosis» del Ministerio de Salud del Perú (MINSA) ^11^. Las muestras pulmonares y extrapulmonares se procesaron dentro de las 24 horas siguientes a su toma.

En la tinción Ziehl-Neelsen, los extendidos se tiñeron con fucsina fenicada al 0,3% y se dejaron teñir durante 5 minutos. A continuación, la decoloración se realizó con solución de alcohol ácido por 3 minutos para luego ser enjuagado con agua para eliminar el exceso de tinción. Posterior a ello, se realizó la coloración de fondo con azul de metileno al 0,1% y se dejó secar al medio ambiente. Los extendidos fueron examinados mediante microscopía directa con un aumento de 100X para identificar los bacilos acido-alcohol resistentes (BAAR).

En la tinción Auramina-Rodamina, la coloración del extendido se realizó con tinción fluorescente y se dejó actuar durante 15 a 20 minutos. La decoloración se realizó con ácido-alcohol durante 1 a 3 minutos, seguido de un aclarado con agua dejando escurrir el exceso de tinción. La coloración de fondo se realizó con permanganato potásico al 0,5% durante 1 minuto y se dejó secar al medio ambiente, protegiéndola de la luz ultravioleta. Los extendidos se examinaron mediante microscopía de fluorescencia con lámpara LED con aumentos de 200X y 400X.

Los resultados de las muestras procesadas fueron ingresados a una base de datos por personal capacitado del laboratorio. Para establecer los criterios de identificación de un resultado positivo en las técnicas de baciloscopia, se siguieron las recomendaciones de la Norma Técnica de Tuberculosis del MINSA ^12^. En la tinción Ziehl-Neelsen, se consideró resultado positivo la presencia de 1 a 9 bacilos BAAR en 100 campos observados (muestra paucibacilar), 10 a 99 BAAR en 100 campos observados, 1 a 10 BAAR por campo en 50 campos observados o 10 BAAR por campo en 20 campos observados. Por otra parte, la tinción Auramina-Rodamina se consideró positiva si, con un aumento de 200X, había de 5 a 49 BAAR en una línea (muestra paucibacilar) o más de 3 BAAR por campo. Mientras que con un aumento de 400X, se consideró un resultado positivo si había entre 3 y 24 BAAR en una línea (muestra paucibacilar) o más de 1 BAAR por campo.

### Pruebas de referencia

Las pruebas de referencia fueron el Xpert MTB/RIF y Xpert Ultra. El procesamiento de las muestras se realizó de acuerdo con las directrices del fabricante (Cepheid Inc., Sunnyvale, CA, EE.UU.) ^13^^,^^14^. Las muestras se almacenaron a una temperatura de 2 a 8 °C. Las muestras se transfirieron a tubos de 15 ml junto con el reactivo proporcionado por el fabricante. Luego los tubos se cerraron y se agitaron enérgicamente de 10 a 20 veces para garantizar una adecuada integración de la muestra. Posterior a ello, se aspiraron entre 500 µL a 2 ml y se colocaron en el cartucho Xpert, que fue insertado en el dispositivo GeneXpert para su procesamiento automatizado.

La diferencia entre Xpert MTB/RIF y Xpert MTB Ultra es el tiempo y la capacidad para detectar diversos genes de *Mycobacterium tuberculosis*. Un resultado positivo para Xpert MTB/RIF se indicó mediante la detección del gen ropβ y sus mutaciones en el umbral de ciclo (Ct), mientras que un resultado positivo para Xpert Ultra se indicó mediante la detección de la secuencia de genes IS6110 e IS1081, independiente de la resistencia a rifampicina ^15^.

### Análisis estadístico

La base de datos se exportó a Microsoft Excel y posteriormente se analizó con el programa estadístico Stata versión 17. Las variables categóricas se presentaron mediante frecuencias absolutas y relativas. La edad de los pacientes se presentó utilizando la mediana y el rango intercuartílico (RIC) debido a la distribución asimétrica de sus datos. Las diferencias en las características de la muestra se evaluaron mediante la prueba Chi cuadrado o prueba exacta de Fisher para variables categóricas y U de Mann-Whitney para la variable edad. En el análisis de rendimiento diagnóstico, se construyeron tablas de doble entrada para calcular la prevalencia, sensibilidad, especificidad, valor predictivo positivo (VPP), valor predictivo negativo (VPN), razón de verosimilitud positiva (RV+) y razón de verosimilitud negativa (RV-) con sus respectivos intervalos de confianza del 95% (IC95%). La prevalencia se calculó como la proporción de casos de tuberculosis definidos como muestras confirmadas por una prueba molecular (Xpert MTB/RIF o Xpert Ultra), en relación con el total de muestras evaluadas. Por último, se trazó la curva de características operativas del receptor (ROC), para calcular el área bajo la curva (AUC) para cada técnica de baciloscopia ^16^.

### Consideraciones éticas

El protocolo del presente estudio fue aprobado por el Comité de Ética de la Facultad de Ciencias de la Salud de la Universidad Privada de Tacna (UPT) con código FACSA-CEI/081-04-2024. Se solicitó la exoneración del consentimiento informado debido a la naturaleza observacional y retrospectiva del estudio. Se registró este protocolo en la plataforma de Proyectos de Investigación en Salud (PRISA) con código EI00000003212, por abordar la temática de tuberculosis en Perú. Adicionalmente, se obtuvo el permiso de la dirección del laboratorio clínico ROE para acceder a la base de datos de tuberculosis. En la recolección y depuración de los datos se garantizó el anonimato de cada individuo.

## RESULTADOS

Se registraron un total de 908 muestras de pacientes con sospecha clínica de tuberculosis, todas las muestras fueron procesadas por tinción Ziehl-Neelsen y 623 de ellas también por tinción Auramina-Rodamina (Figura 1). Asimismo, del total de muestras procesadas, 530 fueron por Xpert MTB/RIF y 378 por Xpert Ultra.

**Figura 1:** Diagrama de flujo del número final de muestras con sospecha de tuberculosis analizadas en el presente estudio.

Las muestras procesadas por Xpert MTB/RIF provinieron de pacientes con una mediana de edad de 60 (RIC 42-74) años. De estas muestras, el 78,3% eran pulmonares, en su mayoría recolectadas por lavado broncoalveolar (38,1%). El 21,7% restante eran muestras extrapulmonares, principalmente de orina (27,8%). Observamos un resultado positivo para tuberculosis en 95 (17,9%) muestras, de los cuales 80 fueron muestras pulmonares y 15 extrapulmonares. Asimismo, del total de muestras positivas para Xpert MTB/RIF, 53 (55,8%) de ellas, no fueron detectadas mediante tinción Ziehl-Neelsen y 41 (53,2%) no fueron detectadas por tinción Auramina-Rodamina (Tabla 1).

**Tabla 1:** Características generales de las muestras procesadas con Xpert MTB/RIF (n=530).

Características		n (%)	Xpert MTB/RIF		
			Positivo n=95 (17,9%)	Negativo n=435 (82,1%)	Valor de p ^a^
Edad (n=395) mediana (RIC)		60 (42-74)	46,5 (35-66)	61 (46-75)	<0,001
Género (n=466)					0,277
	Femenino	247 (53,0)	39 (15,8)	208 (84,2)	
	Masculino	219 (47,0)	43 (19,6)	176 (80,4)	
Origen de la muestra					0,123
	Pulmonar	415 (78,3)	80 (19,3)	335 (80,7)	
	Extrapulmonar	115 (21,7)	15 (13,0)	100 (87,0)	
Muestras pulmonares					0,337
	Lavado broncoalveolar	158 (38,1)	30 (19,0)	128 (81,0)	
	Aspiración bronquial	105 (25,3)	25 (23,8)	80 (76,2)	
	Esputo	152 (36,6)	25 (16,5)	127 (83,5)	
Muestras extrapulmonares					0,026
	LCR	21 (18,3)	3 (14,3)	18 (85,7)	
	Orina	31 (27,8)	1 (3,1)	31 (96,9)	
	Líquido pleural	17 (14,8)	2 (11,8)	15 (88,2)	
	Liquido ascítico	4 (3,5)	0 (0,0)	4 (100,0)	
	Biopsia de ganglio linfático	5 (4,3)	3 (60,0)	2 (40,0)	
	Biopsia de tejidos blandos y parénquima	11 (9,6)	1 (9,1)	10 (90,9)	
	Absceso de tejidos blandos y parénquima	12 (10,4)	1 (8,3)	11 (91,7)	
	Otros	13 (11,3)	4 (30,8)	9 (69,2)	
Tinción Ziehl-Neelsen (n=530)					< 0,001
	Positivo	45 (8,5)	42 (93,3)	3 (6,7)	
	Negativo	485 (91,5)	53 (10,9)	432 (89,1)	
Tinción Auramina-Rodamina (n=395)					< 0,001
	Positivo	36 (9,1)	36 (100,0)	0 (0,0)	
	Negativo	359 (90,9)	41 (11,4)	318 (88,6)	

RIC: rango intercuartílico, LCR: líquido cefalorraquídeo

a El valor p para las variables categóricas se calcularon mediante la prueba chi-cuadrado o prueba exacta de Fisher (en muestras extrapulmonares). El valor p para la variable edad del paciente se calculó mediante la prueba U de Mann-Whitney.

Las muestras procesadas con Xpert Ultra, provinieron de pacientes con una mediana de edad de 56 (RIC 37-74) años, siendo más de la mitad de sexo masculino (54,3%). De estas muestras procesadas, el 78,3% eran de origen pulmonar, principalmente de aspiración bronquial (40,9%). Las muestras con un resultado positivo fueron 104 (27,5%), de los cuales 91 eran de origen pulmonar y 13 de origen extrapulmonar. Del mismo modo, del total de muestras positivas para Xpert Ultra, 76 (73,1%) de ellas no fueron identificados con tinción Ziehl-Neelsen y 42 (70,0%) tampoco fueron detectados con tinción Auramina-Rodamina (Tabla 2).

**Tabla 2:** Características generales de las muestras procesadas con Xpert Ultra (n=378).

Características		n (%)	Xpert Ultra		
			Positivo n=104 (27,5%)	Negativo n=274 (72,5%)	Valor de p ^a^
Edad (n=335), mediana (RIC)		56 (37-74)	48 (30-69)	59 (41-75)	0,006
Genero (n=328)					0,448
	Femenino	150 (45,7)	39 (26,0)	111 (74,0)	
	Masculino	178 (54,3)	53 (29,8)	125 (70,2)	
Origen de la muestra					0,008
	Pulmonar	296 (78,3)	91 (30,7)	205 (69,3)	
	Extrapulmonar	82 (21,7)	13 (15,8)	69 (84,2)	
Muestras pulmonares					0,213
	Lavado broncoalveolar	85 (28,7)	32 (37,7)	53 (62,3)	
	Aspiración bronquial	121 (40,9)	36 (29,8)	85 (70,2)	
	Esputo	90 (30,4)	23 (25,6)	67 (74,4)	
Muestras extrapulmonares					0,192
	LCR	6 (7,3)	0 (0,0)	6 (100,0)	
	Orina	20 (24,4)	3 (15,0)	17 (85,0)	
	Líquido pleural	20 (24,4)	1 (5,0)	19 (95,5)	
	Liquido ascítico	4 (4,9)	0 (0,0)	4 (100,0)	
	Biopsia de ganglio linfático	7 (8,5)	3 (42,9)	4 (57,1)	
	Biopsia de tejidos blandos y parénquima	5 (6,1)	1 (20,0)	4 (80,0)	
	Absceso de tejidos blandos y parénquima,	11 (13,4)	2 (18,2)	9 (81,8)	
	Otros	9 (11,0)	3 (33,3)	6 (66,7)	
Tinción Ziehl-Neelsen (n=378)					< 0,001
	Positivo	32 (8,5)	28 (87,5)	4 (12,5)	
	Negativo	346 (91,5)	76 (22,0)	270 (78,0)	
Tinción Auramina-Rodamina (n=228)					< 0,001
	Positivo	21 (9,2)	18 (85,7)	3 (14,3)	
	Negativo	207 (90,8)	42 (20,3)	165 (79,7)	

RIC: rango intercuartílico, LCR: líquido cefalorraquídeo

a El valor p para las variables categóricas se calcularon mediante la prueba chi-cuadrado o test exacto de Fisher (en muestras extrapulmonares). El valor p para la variable edad del paciente se calculó mediante la prueba U de Mann-Whitney.

Al comparar la tinción Ziehl-Neelsen con la tinción Auramina-Rodamina, se observó una buena concordancia entre las dos técnicas de baciloscopia (índice Kappa de Cohen=0,990). Observamos que solo una muestra positiva con la tinción Ziehl-Neelsen no se detectó en la tinción Auramina-Rodamina. Por otra parte, se observó una concordancia moderada al comparar la tinción Ziehl-Neelsen (índice Kappa de Cohen=0,548) y la tinción Auramina-Rodamina (índice Kappa de Cohen=0,586) con Xpert MTB/RIF, y una concordancia débil al comparar ambas técnicas de baciloscopia con Xpert Ultra (índice Kappa de Cohen=0,324 y 0,357) (Tabla 3).

**Tabla 3:** Análisis de concordancia de las técnicas de baciloscopia comparadas con Xpert MTB/RIF y Xpert Ultra.

Comparación de pruebas	Concordancia global	Índice de Kappa Cohen	Error estándar	Valor de p
Tinción Ziehl-Neelsen vs. tinción Auramina-Rodamina	99,8	0,990	0,040	< 0,001
Tinción Ziehl-Neelsen vs. Xpert MTB/RIF	89,4	0,548	0,039	< 0,001
Tinción Ziehl-Neelsen vs. Xpert Ultra	78,8	0,324	0,041	< 0,001
Tinción Auramina-Rodamina vs. Xpert MTB/RIF	89,6	0,586	0,046	< 0,001
Tinción Auramina-Rodamina vs. Xpert Ultra	80,3	0,357	0,055	< 0,001

Al tomar como referencia al Xpert MTB/RIF, encontramos que, para el total de muestras, el rendimiento diagnóstico de la tinción Ziehl-Neelsen (AUC=0,72; IC95%: 0,67-0,77) y la tinción Auramina-Rodamina (AUC=0,73; IC95%: 0,68-0,79) eran similares (Material suplementario 1A y 1B ).

Asimismo, reportamos una sensibilidad del 44,2% (IC95%: 34,0-54,8) y del 46,8% (IC95%: 35,3-58,5), y una especificidad del 99,3% (IC95%: 98,0-99,9) y del 100% (IC95%: 98,8-100), respectivamente (Material suplementario 2A). Se observó una diferencia en la capacidad diagnóstica en las muestras extrapulmonares, mostrando la tinción Auramina-Rodamina un mejor rendimiento diagnóstico (AUC=0,71; IC95%: 0,52-0,91) en comparación con la tinción Ziehl-Neelsen (AUC=0,65; IC95%: 0,53-0,78) (Tabla 4).

**Tabla 4:** Rendimiento diagnóstico de las técnicas de baciloscopia para la tuberculosis pulmonar y extrapulmonar en comparación con Xpert MTB/RIF.

	Prevalencia % (IC95%)	Sensibilidad % (IC95%)	Especificidad % (IC95%)	VPP (IC95%)	VPN (IC95%)	RV + (IC95%)	RV - (IC95%)	AUC/ROC (IC95%)
Muestras totales								
Tinción Ziehl-Neelsen	17,9 (14,8-21,5)	44,2 (34,0-54,8)	99,3 (98,0-99,9)	93,3 (81,7-98,6)	89,1 (86,0-91,7)	64,1 (20,3-202,5)	0,6 (0,5-0,7)	0,72 (0,67-0,77)
Tinción Auramina-Rodamina	19,5 (15,7-23,7)	46,8 (35,3-58,5)	100 (98,8-100)	100 (90,3-100)	88,6 (84,8-91,7)	-	0,5 (0,4-0,7)	0,73 (0,68-0,79)
Muestras pulmonares								
Tinción Ziehl-Neelsen	19,3 (15,6-23,4)	46,3 (35,0-57,8)	100 (98,9-100)	100 (90,5-100)	88,6 (85,0-91,6)	-	0,5 (0,4-0,7)	0,73 (0,68-0,79)
Tinción Auramina-Rodamina	18,8 (15,0-23,2)	47,1 (35,1-59,4)	100 (98,8-100)	100 (89,4-100)	89,1 (85,3-92,2)	-	0,5 (0,4-0,7)	0,74 (0,68-0,79)
Muestras extrapulmonares								
Tinción Ziehl-Neelsen	13,0 (7,5-20,6)	33,3 (11,8-61,6)	97,0 (91,5-99,4)	62,5 (24,5-91,5)	90,7 (83,5-95,4)	11,1 (2,9-41,8)	0,7 (0,5-0,8)	0,65 (0,53-0,78)
Tinción Auramina-Rodamina	30,4 (13,2-52,9)	42,9 (9,9-81,6)	100 (79,4-100)	100 (29,2-100)	80,0 (56,3-94,3)	-	0,6 (0,3-1,1)	0,71 (0,52-0,91)

AUC: área bajo la curva; ROC: características operativas del receptor; VPP: valor predictivo positivo; VPN: valor predictivo negativo; RV: razón de verosimilitud; IC95%: intervalo de confianza del 95%.

Al tomar como referencia al Xpert Ultra, el rendimiento diagnóstico para muestras totales fue de AUC=0,63 (IC95%: 0,58-0,67) para la tinción Ziehl-Neelsen (Material suplementario 1C), con una sensibilidad y especificidad del 26,9% (IC95%: 18,7-36,5) y 98,5% (IC95%: 96,3-99,6), respectivamente (Material suplementario 2B). El rendimiento diagnóstico de la tinción Auramina-Rodamina fue de AUC=0,64 (IC95%: 0,58-0,70) con una sensibilidad del 30% (IC95%: 18,8-43,2) y especificidad del 98,2% (IC95%: 94,9-99,6) (Material suplementario 1D y 2B). En las muestras pulmonares, encontramos una ligera diferencia en el rendimiento diagnóstico entre la tinción Auramina-Rodamina (AUC=0,65;IC95%%: 0,59-0,71) y la tinción Ziehl-Neelsen (AUC=0,63; IC95%%: 0,58-0,68) (Tabla 5).

**Tabla 5:** Rendimiento diagnóstico de las técnicas de baciloscopia para la tuberculosis pulmonar y extrapulmonar en comparación con Xpert Ultra.

	Prevalencia % (IC95%)	Sensibilidad % (IC95%)	Especificidad % (IC95%)	VPP (IC95%)	VPN (IC95%)	RV + (IC95%)	RV - (IC95%)	AUC/ROC (IC95%)
Muestras totales								
Tinción Ziehl-Neelsen	27,5 (23,2-32,3)	26,9 (18,7-36,5)	98,5 (96,3-99,6)	87,5 (71,0-96,5)	78,0 (73,3-82,3)	18,4 (6,6-51,3)	0,7 (0,7-0,8)	0,63 (0,58-0,67)
Tinción Auramina-Rodamina	26,3 (20,7-32,5)	30,0 (18,8-43,2)	98,2 (94,9-99,6)	85,7 (63,7-97,0)	79,7 (73,6-85,0)	16,8 (5,1-55,0)	0,7 (0,6-0,8)	0,64 (0,58 -0,70)
Muestras pulmonares								
Tinción Ziehl-Neelsen	30,7 (25,5-36,3)	27,5 (18,6-37,8)	98,5 (95,8-99,7)	89,3 (71,8-97,7)	75,4 (69,8-80,4)	18,8 (5,8-60,6)	0,7 (0,7-0,8)	0,63 (0,58-0,68)
Tinción Auramina-Rodamina	26,5 (20,7-32,9)	31,6 (19,9-45,2)	98,1 (94,6-99,6)	85,7 (63,7-97,0)	79,9 (73,6-85,3)	16,6 (5,1-54,4)	0,7 (0,6-0,8)	0,65 (0,59-0,71)
Muestras extrapulmonares								
Tinción Ziehl-Neelsen	15,9 (8,7-25,6)	23,1 (5,0-53,8)	98,6 (92,2-100,0)	75,0 (19,4-99,4)	87,2 (77,7-93,7)	15,9 (1,8-141,5)	0,8 (0,6-1,1)	0,61 (0,49 -0,73)
Tinción Auramina-Rodamina^a^	-	-	-	-	-	-	-	-

AUC: área bajo la curva; ROC: características operativas del receptor; VPP: valor predictivo positivo; VPN: valor predictivo negativo; RV: razón de verosimilitud; IC95%: intervalo de confianza del 95%.

a Sólo 13 muestras extrapulmonares fueron procesadas con Auramina-Rodamina, todas resultando negativas para TB extrapulmonar.

## DISCUSIÓN

Se evaluó el rendimiento diagnóstico de la tinción Ziehl-Neelsen y Auramina-Rodamina, utilizando como referencia pruebas moleculares recomendadas por la OMS (Xpert MTB/RIF y Xpert Ultra). Nuestros hallazgos indican que ambas técnicas de baciloscopia tienen un rendimiento diagnóstico moderada en la identificación de casos de tuberculosis.

El rendimiento diagnóstico puede variar en función del patrón de referencia utilizado. En estudios anteriores sobre el diagnóstico de la tuberculosis, el cultivo de micobacterias fue el patrón de oro, generando una buena capacidad discriminativa en ambas técnicas de baciloscopia (AUC=89,7 y 90,3) y una sensibilidad superior al 60% ^17^. Sin embargo, las desventajas del cultivo como el tiempo de espera para el crecimiento micobacteriano (2 a 3 semanas) y la necesidad de laboratorios con nivel de bioseguridad III para analizar muestras de microorganismos que provocan enfermedades graves, dificultan su uso como método de referencia ^18^^,^^19^. Por lo tanto, es necesario establecer un estándar de referencia que supere estas barreras evidenciadas, siendo una opción factible el uso de pruebas moleculares.

En muestras pulmonares, observamos un regular rendimiento diagnóstico en ambas técnicas de baciloscopia, con una ligera ventaja en la capacidad discriminativa de la tinción Auramina-Rodamina sobre la tinción Ziehl-Neelsen. Estos resultados concuerdan con los de Dzodanu *et al*., quienes destacaron un mayor rendimiento diagnóstico de las técnicas de fluorescencia ^8^. Estas técnicas permiten una rápida identificación de los bacilos en un campo de fondo oscuro, lo que reduce el tiempo necesario para reportar los resultados ^20^.

Otro aspecto importante del estudio de Dzodanu *et al*. es el mayor rendimiento diagnóstico observada en ambas técnicas de baciloscopia (AUC=0,92 y 0,77) en comparación con nuestros resultados ^8^. Esta discrepancia se hace más evidente al analizar los resultados obtenidos con un patrón de referencia diferente. Mientras que Dzodanu *et al*. utilizaron Xpert MTB/RIF, nosotros utilizamos Xpert Ultra. Según investigaciones anteriores, Xpert Ultra es la prueba molecular con mejor sensibilidad (entre el 86% y el 100%) y especificidad (entre el 89% y el 99%), por lo que debería considerarse como estándar de oro para estudios de diagnóstico de tuberculosis pulmonar ^5^.

En las muestras extrapulmonares, observamos que las tinciones Ziehl-Neelsen y Auramina-Rodamina presentaron una capacidad discriminativa moderada, pero una baja sensibilidad de 33,3% y el 42,9%, respectivamente. La sensibilidad y especificidad de las técnicas de baciloscopia suelen ser inferiores en comparación con las pruebas moleculares ^9^. Xpert MTB/RIF y Xpert Ultra pueden ser útiles para diagnosticar tuberculosis extrapulmonar; sin embargo, los parámetros diagnósticos varían en función del tipo de muestra ^21^. Se reportó que ambos métodos moleculares tuvieron una sensibilidad variable para muestras de líquido cefalorraquídeo (71% y 89%), liquido pleural (50% y 75%) y aspirado de ganglios linfáticos (82% y 70%); mientras la especificidad fue más del 85% en los tres tipos de muestra ^22^. Por lo tanto, es necesario elegir el método diagnóstico en función del tipo de muestra extrapulmonar que se pretende evaluar.

Nuestros resultados reportan que las técnicas de baciloscopia tienen una capacidad inadecuada para diagnosticar tuberculosis. En muchos países de bajos y medianos ingresos se sigue usando la baciloscopia como método diagnóstico inicial ^23^. La principal desventaja de estas técnicas convencionales es la deficiente sensibilidad, que impide identificar correctamente a todos los pacientes con tuberculosis pulmonar o extrapulmonar, produciendo un gran porcentaje de falsos negativos. La OMS recomienda el uso de pruebas moleculares que tienen un mejor rendimiento diagnostico como el Xpert MTB/RIF o Xpert Ultra, sin embargo, su costo de mantenimiento limita su implementación en diversos países ^24^. Una alternativa es el uso combinado de técnicas de baciloscopia con métodos moleculares, donde solo aquellas muestras con resultado negativo en las técnicas de baciloscopia puedan ser procesadas por pruebas moleculares ^25^, sin embargo, la limitada cantidad de laboratorios públicos en el Perú retrasaría el diagnóstico.

Otra alternativa es el uso de algoritmos diagnósticos. Un estudio realizado en Lima, Perú, informó que la combinación de radiografía de tórax seguido de una prueba molecular alcanzó una sensibilidad del 68%, en comparación con el enfoque basado en la evaluación sintomática seguida de microscopia de frotis, cuya sensibilidad fue de solo 23% ^26^. Los algoritmos que excluyen la evaluación clínica radiográfica y dependen únicamente de pruebas bacteriológicas con bajo rendimiento diagnóstico como la baciloscopia, pueden ser percibidos como menos costosos. Sin embargo, el diagnóstico inadecuado y la falta de tratamiento oportuno pueden generar mayores costos en el sistema de salud a largo plazo ^27^.

Asimismo, el procesamiento de muestras agrupadas es una alternativa accesible para la implementación de pruebas moleculares. Se observó que el análisis de muestras agrupadas mediante Xpert MTB/RIF o Xpert Ultra tiene una sensibilidad del 95% y una especificidad del 97,1%, además alcanza una concordancia de hasta el 96% con los resultados obtenidos en el procesamiento de muestras individuales ^28^. Del mismo modo, el procesamiento de muestras agrupadas representa un menor costo en comparación con el análisis de muestras individuales, siendo una opción factible y confiable para países de bajos y medianos ingresos, como Perú ^29^.

Nuestro estudio presenta algunos elementos que deben ser considerados al momento de interpretar los resultados. Por una parte, si bien existen muchos estudios que comparan la baciloscopia con técnicas moleculares como el Xpert MTB/RIF o Xpert Ultra, nuestro trabajo fue desarrollado en un contexto particular, un laboratorio privado en el cual la mayoría de las muestras no eran esputo sino muestras de aspirado bronquial y lavado broncoalveolar, lo cual genera información sobre este contexto en particular. Por otro lado, hay que reconocer las limitaciones como no contar con información detallada sobre las características sociodemográficas, antecedentes de tuberculosis, comorbilidades o tratamiento previo de los pacientes. Estos factores pueden influir en el desempeño de las pruebas diagnósticas, lo que podría generar un sesgo de medición. Segundo, la distribución de muestras pulmonares y extrapulmonares no fue uniforme, y como ha sido mencionado, el contexto particular en el que fue desarrollado impide generalizar los hallazgos a otros escenarios. Tercero, el número limitado de muestras extrapulmonares impidió determinar el rendimiento diagnóstico para cada tipo de muestra. Por último, los resultados pueden estar sujetos a sesgos derivados de errores en el procesamiento de las muestras o relacionados a la interpretación de resultados por parte del personal de laboratorio. A pesar de estas limitaciones, las muestras fueron recolectadas y analizadas en un laboratorio clínico privado con certificación de calidad, utilizando equipos modernos que garantizan resultados confiables. Además, este estudio evalúa el rendimiento diagnóstico de técnicas de baciloscopia tomando como referencia pruebas moleculares recomendadas por la OMS, las cuales presentan una mayor capacidad diagnóstica que el cultivo micobacteriano.

En conclusión, se observó que la tinción de Ziehl-Neelsen y la tinción Auramina-Rodamina tienen un rendimiento diagnóstico moderado para muestras pulmonares y extrapulmonares. Al comparar los resultados de ambas técnicas de microscopía, observamos una capacidad diagnóstica ligeramente superior con la tinción de Auramina-Rodamina. Además, ambas tinciones mostraron una excelente especificidad, pero una baja sensibilidad. Por lo tanto, se recomienda evaluar la implementación de nuevas alternativas diagnósticas, que incluyan el uso de pruebas moleculares de procesamiento rápido y rendimiento diagnóstico adecuado para detectar tuberculosis, previa evaluación de los costos asociados.
